# Short-Term and Medium-Term Impact of Retirement on Sport Activity, Self-Reported Health, and Social Activity of Women and Men in Poland

**DOI:** 10.1155/2019/8383540

**Published:** 2019-04-11

**Authors:** Elżbieta Biernat, Łukasz Skrok, Justyna Krzepota

**Affiliations:** ^1^Department of Tourism, Collegium of World Economy, SGH Warsaw School of Economics, al. Niepodległości 162, 02-554 Warsaw, Poland; ^2^Department of Business Economics, Collegium of World Economy, SGH Warsaw School of Economics, al. Niepodległości 162, 02-554 Warsaw, Poland; ^3^Department of Physical Culture and Health Promotion, University of Szczecin, al. Piastów 40B blok 6, 71-065 Szczecin, Poland

## Abstract

*Background. *The aim of this study was to assess how retirement affects the short-term (up to 2 years after retirement) and medium-term (2-4 years) sport/exercise activity (S/EA) of Poles. To gain a broader context for interpretation, the effect of retirement on self-rated health status, attitudes, social activity, and sexual life was analysed.* Methods.* A quasiexperiment utilizing data from the longitudinal study Social Diagnosis and radius-matching method was conducted. Retiring men and women were matched with similar, nonretiring ones to make comparisons of differences in S/EA and other outcomes interpretable in terms of causality.* Results.* Retirement does not have a significant effect in the short term on S/EA of men (p=.440) and women (p=.340). The satisfaction of men with their health status in this period was improved (p=.007), although they more often declared health problems that impaired their everyday functioning (p=.045). Women rarely reported serious health problems (p=.024). In the medium perspective, retirement had the effect on reducing S/EA in men (p=.012) and various dimensions of their social life. Although men tend to worry more often about their health (p<0.001), they are less likely to suffer from problems with moving (p=.001) and fatigue (p=.013). Despite the fact that women are more often satisfied with their health (p=.027), they also more often complain about heart or chest pain (p=.010), body pain (p=.009), and fatigue (p=.007).* Conclusion*. It is necessary to prepare employees for retirement much earlier than in the preretirement age. In addition to raising awareness of the effect of S/EA functions, it is necessary to monitor the physical activity of employees and to use appropriate programmes for (1) maintaining motivation among employees who are physically active before they retire and (2) raising awareness and encouraging physical activity in employees who are physically passive.

## 1. Introduction

The demographic landscape in Poland (similar to that all over the world) is changing dynamically. Older adults represent the fastest growing group of the general population [1]. At the end of 2016, the number of Poles of postworking age (men ≥65 years, women ≥60 years) was 7,770 thousand, with its share exceeding a fifth of the general population. Compared to 2015, this number has increased by 236 thousand (6%). The World Health Organization [2] warns that* the proportion of people aged 60 and over is growing faster than any other age group. Between 1970 and 2025, a growth in older persons of some 694 million or 223 percent is expected. In 2025, there will be a total of about 1.2 billion people over the age of 60. By 2050 there will be 2 billion, with 80 percent of them living in developing countries*. This growing problem requires the introduction of new economic, social and health solutions, allowing for the best possible utilization of human capital in the postworking age. However, it is not only economic effects that matter. Also important is the respective functioning of older people in society, and their well-being in physical, social, and mental terms, in other words active ageing [2], which is closely related to the ability to continue to perform life-related activities.

According to researchers, sport/exercise activity (S/EA) is critical in the process of active ageing [3–6], particularly if physical and functional autonomy can be maintained throughout ageing, thus minimizing degeneration and improving health and the quality of life (QoL) [7]. This is consistent with the findings of Meisner and Baker [8] and Siqueira et al. [9], who argued that physical activity (PA) is a way of improving organic conditions and slowing physical degeneration. Adapted exercise programmes can slow down the decline in health-related quality of life among elderly persons [10–13]. Performed exercises delay the processes of dementia and the course of Alzheimer's disease [14] and produce an increased range of motion, muscle strength, and functional autonomy levels [15, 16]. Watsford et al. [17] demonstrated the reduced capacity of the respiratory system with ageing and described the effect that habitual physical activity has upon this decline. Therefore, it is unsurprising that the correlation between PA and QoL is a subject of research [18] and that regularly exercising seniors declare a higher subjective self-reported health status and improved moods, from the standpoint of both physical and mental health, and enjoy a better QoL [19]. What is surprising is the fact that, in general, seniors are rarely involved in PA [20]. Therefore, the aim of the present analysis was to assess how retirement affects the sport/exercise activity (S/EA) of men and women in Poland, using both the short-term perspective (up to two years after retirement, from March/April 2011 to March/April 2013) and medium-term perspective (from two to four years after retirement, i.e., until March/April 2015). Furthermore, in order to gain a context for interpretation, the impact retirement has on self-rated health, attitudes to life, social activity (measured, among other things, with the level of participation in various forms of social life and the declared number of friends), and satisfaction with sexual life was also measured. The motivation behind the scope of this research was that the combined analysis of individual aspects of life may allow for the incorporation of both social and physical motivations behind S/EA into the interpretation.

## 2. Material and Methods

Conducted analysis utilizes* Social Diagnosis* data.* Social Diagnosis* was a longitudinal study on the conditions and quality of life in Poland. This extensive survey was conducted periodically from 2000 to 2015 (for the most part, biannually). Since the question about S/EA in its final, full version (Do you practice any sport or physical activity?: (1) no, I do not practice any sport or physical activity; (2) aerobics; (3) running/jogging/Nordic walking; (4) gym; (5) cycling; (6) skiing or other winter sports; (7) swimming; (8) football or other team sports; (9) yoga; (10) martial arts; (11) another sport or type of physical activity) was only asked from 2011, only data from 2011, 2013, and 2015 were used within the analysis. In each year, the survey was conducted in March or in the first half of April.* Social Diagnosis* was conducted concurrently on two levels: households and individuals (the latter among individuals at least 16 years old). Households were selected using a two-stage (statistical regions and dwellings) stratified (by voivodeship and size of the place of residence) sampling method. Within the analysis, information on individuals examined in 2011 (when 26,453 individuals were examined), 2013 (when 26,308 individuals were examined), and 2015 (when 24,324 individuals were examined) was used. In 2013, 68% of the individuals examined in 2011 remained (n=18,020), whereas in 2015: 67% of 2013 (n=17,498) and 43% of 2011 (n=11,461) [21]. A complete dataset, along with a detailed description, questionnaires, and instructions, is available, both in English and Polish, at www.diagnoza.com. [22]. A detailed description of the variables used, as well as descriptive statistics, is included in the annexes.

## 3. Estimation Method

The Radius Matching on Propensity Score with Bias Adjustment [23, 24] was adopted as a method for the conducted analysis. The method, applied to a longitudinal data, allowed for causal analysis of the start of retirement and S/EA. In this respect, our analysis follows the one by Behncke [25], who, by utilizing the radius-matching method to analyse the English Longitudinal Study of Ageing, found significant, negative health effects of retirement. The Stata Statistical Software: Release 15 [26] was used, along with radius match package authored by Huber et al. [24].

According to the fundamental assumption behind the matching method, whenever two individuals had the same ex ante probability of retiring, but only one of them indeed retired, this event was random. Therefore, the analysis is of a pseudoexperimental nature and the difference in chosen outcomes in the following fates of the pair can be interpreted as effects of retiring (if occurring repeatedly among numerous such pairs; formally, whether the difference in outcomes between retired and nonretired is statistically different from zero).

Firstly, a probit model allowing for calculation of probability of retiring between 2011 and 2013 was estimated, separately for men and women 48-65 years old (when filling the 2011 survey). Both genders constituted separate strata, for which the entire estimation procedure was conducted independently. The sample comprised 2,730 women and 2,880 men who had not been retired when surveyed in 2011. The retirement was defined as a declaration of the retirement benefit being a main source of income with no supplementary income from work in any form (including running owns business or assisting in running one). Even though the retirement age in Poland (during the period covered by the analysis) was 60 for women and 65 for men, qualifying for a retirement benefit at an earlier age was not uncommon in Poland. For example, according to the Central Statistical Office of Poland (Główny Urząd Statystyczny, GUS), there were close to 721,000 men and 421,000 women in 2011 [27] (Since age of 50 (as of 2013) was the lowest age, for which one could say that at least 1 woman and at least 1 man of such an age, and of any higher, in the Social Diagnosis sample was retired in 2013, 50 was chosen as the bottom limit for our subsample.). As a treatment variable, being retired in 2013 was used. As covariates in the probit models a set of demographic, economic (including a profession, which is crucial for the possibility of early retirement), social, psychophysical variables based on 2011 survey were used (Due to size of the tables – the model comprises almost 200 covariates – they were omitted from the article. They can be made available upon request. For women, chi^2^ for the Wald test is 546.74 (p<.001) and the pseudo-R^2^ is .366. After omitting observations with missing data and with occupations perfectly determining retirement, 2,175 observations were used. For men, the corresponding values are: chi^2^ = 343.87 (p<.001), pseudo-R^2^ = .3938, n=2,012.).

After eliminating outliers (in line with the procedure proposed by Huber et al. [24]), the number of observations used (depending on the outcome variable) was between 1,405 and 1,428 for men and between 1,172 and 1,190 for women, for outcome variables measured in 2013, as well as between 920 and 929 for men and between 781 and 798 for women, for outcome variables measured in 2015. These groups were divided into the treated (i.e., people, who were retired in 2013) and nontreated (i.e., people, who were not retired in 2013).

In the next step, treated people were matched with hypothetical, averaged people (benchmarks), each based on nontreated people of the same gender and as similar as possible. In other words, the characteristics of the benchmarks were calculated based on characteristics of the real nontreated people included in the sample which were similar enough to the treated ones. The more similar the former were to the latter, the greater was the weight assigned to them when calculating the average. For outcomes measured in 2013, there are between 154 and 157 such pairs (consisting of real treated and hypothetical nontreated people) for men and between 218 and 222 for women and for outcomes measured in 2015 between 87 and 88 for men and between 160 and 164 for women.

Analogously, nontreated people were matched with treated benchmarks. The latter were calculated as hypothetical, averaged entities based on real treated people, similar enough to the nontreated people in question. For outcomes measured in 2013, there are between 1,251 and 1,271 such pairs (consisting of hypothetical treated and real nontreated people) for men and between 954 and 968 for women and for outcomes measured in 2015 between 833 and 841 for men and between 618 and 634 for women.

In line with fundamental assumption of matching estimation, pairs (both for treated and nontreated people) were matched using theoretical probabilities of retiring between 2011 and 2013, calculated with probit models from the first step. However, matching was further based on balancing variables considered especially important in the context of either retiring or monitored outcomes. These following continuous and ordinal variables were used: age, lust for life (with 1-10 scale), BMI, number of adults living in the same household, number of friends, number of acquaintances met regularly, dissatisfaction from health (with 1-6 scale), and number of years in education system. Furthermore, the following dummy variables were used as balancing variables as well: marital status, sport activity, higher education, permanent employment, inactive, living in a large city, living in a rural area, higher education of father, problems with sleeping, high energy, health benefits, general trust, and trust towards parliament (all measured in 2011). Additionally, for each effect, a corresponding variable based on data from 2011 (if not within the “general” set of balancing variables) was included as a balancing variable (except for membership in sports club, which was not subject of a question in 2011). Technically, the similarity was calculated using the Mahalanobis distance. The values of parameters (radius, weights) used within the estimation are the default ones for the radius match package.

In the third step average values of outcome variables, measured both in 2013 and in 2015, were calculated, separately for the treated people (and their benchmarks) and for the nontreated ones (and their benchmarks). These variables include S/EA and membership of sports clubs, but also—for the extended analytical context—health variables (too much alcohol in previous year, dissatisfaction with health, BMI), attitude (willingness to live), social activity (friends met regularly, acquaintances met regularly, number of friends, work for local society, member of organizations, took part in public meeting, voluntary activities), and sexual activity (dissatisfaction with sex life, losing interest in sex).

Calculated values were adjusted with bias adjustment procedure proposed by Huber et al. [24] to decrease the bias resulting from eventual differences in distributions of the propensity score and the balancing variables between the treated and the nontreated.

The differences between average values of the outcome variables for the treated and their benchmarks constitute the Average Treatment Effects on the Treated (ATETs), and the differences between average values of the outcome variables for the nontreated and their benchmarks constitute the Average Treatment Effects on the nontreated (ATENTs). Tables 1 and 2 contain Average Treatment Effects (ATEs) for women and men, which are the averaged ATETs and ATENTs. In other words, ATE is an average of an effect of retirement for people who have retired between 2011 and 2013 (ATET) and hypothetical effect of retirement for people who have not (ATENT). For calculation of ATE, weights are calculated based on the number of people in the treated (weight for the ATET) and nontreated groups (weight for the ATENT). When describing results measured by dummy variables, the outcomes represent differences between frequencies of positive answers in the two compared groups (measured in percentage points, abbreviated as pp).

## 4. Results

Short-run effects of retirement (taking place between 2011 and 2013) can be seen in Table 1, separately for women and men. They show that retiring has no significant impact on the S/EA of either men (p=.44) or women (p=.34). Nevertheless, the results suggest that that membership of retired women in sports clubs decreases by .3 pp (p=.06) when comparing with nonretired women.

However, the impact of retirement on social activities is different. In particular, there is no significant effect on women. For retired men, the number of met acquaintances is lower compared to nonretired ones (by 1.3 people; p=.011), as well as number of organizations to which they belong (almost by half; p=.046). Lower social activity of retired men is accompanied by their lower lust for life (by .4 points, with the variable being a 1-10 ordinal variable; p=.013), while the effect is reversed for retired women (increase by .3 points; p=.021).

The analysis of health outcomes shows mixed results for men and weak, but rather positive, for women. Compared to nonretired men, satisfaction from one's own health increases (by .3 with the variable being a 1-6 ordinal variable; p=.007), while BMI of retired men decreases (by 1.4 points; p<0.001). For women such an effect cannot be observed. Retired men complain about problems with sleeping less often (by 8 pp; p=.004) and body pains (by 8 pp; p=.058). On the other hand, retirement means an increase in propensity of men to declare drinking too much alcohol in the previous year (more than 2.5 times; p<0.001), whereas for retired women it is nonexisting (while 1.7% nonretired women declares so; p<0.001). More retired men than nonretired ones declare constant worrying about their health (by 10 pp; p=.009) and due to digestive problems (by 8 pp; p=.041). Furthermore, greater number of retired men complain about often health problems that hamper their everyday life (by 7 pp; p=.045), but lower number of them complain about having such problems, ‘once or twice in recent months' (by 15 pp; p=.001). Moreover, greater fractions of retired men declare being seriously ill in the previous year (by 8 pp; p=.022), while for women, the opposite effect occurs (by 6 pp; p=.024). Men tend to suffer from palpitation more often when retired (by 10 pp; p=.012), and women less often (by 7 pp; p=.037). Fewer number of both retired women (by 6 pp; p=.048) and men (by 14 pp; p<0.001) have problems with sweating.

In the middle term (within the following two to three years), the outcome of retiring is a lower S/EA of men (by 12 pp; p=.012; Table 2). Importantly, this effect is a result of the prior (i.e., in 2011) greater propensity to exercise by the men who retired afterwards (32.4% of the men who retired engaged in S/EA in 2011, before retirement, while only 25.8% of the men who did not retire between 2011 and 2013 engaged in S/EA). In time, the fraction of men being active decrease for both retired and nonretired, but the initial difference between them narrows down as well (in 2015, 26.8% of the men who retired between 2011 and 2013 engaged in S/EA, while 23.5% of these who did not retire in the same period were). In other words, it seems that, for some of the retiring men, the unusually high occurrence of S/EA was work-related and mostly disappeared after retiring.

A negative impact of retirement on BMI of men is still visible (which suggests decrease in weight; p<.005). Moreover, one can observe negative impact on membership in sports clubs (nonexistent for the retired, with .8% membership ratio for the nonretired; p=.008) as well as participation in public meetings (importantly, meetings in the workplace are irrelevant; by 12 p.p; p=.012) and number of acquaintances met (by 1.7 people; p=.015). There is also a hint of a negative impact of retirement on voluntary activities (by 10 pp; p=.059). Probability of losing interest in matters of sex decreases (by 27 pp; p<.005), but dissatisfaction with sex life increases (by .36; p=.07). For women, losing interest in matters of sex takes place more often when retired (by 7.1 pp; p=.005).

When it comes to health behaviour of retired women one should pay attention to lack of indication of overusing alcohol in the previous year (while 2% of nonretired women declared so; p<0.001), positive impact on the BMI (by .85; p=.044), and negative on dissatisfaction from one's health (by .24; p=.027). In comparison with nonretired women, the retired ones complain less often about stomach pains (by 10 pp; p=.015), but more often about chest or hearth pains (by 11 pp; p=.010), body pains (by 11 pp; p=.009), palpitation (by 9 pp; p=.030), and tiredness not related to work (by 12 pp; p=.007).

In case of men, some of the negative short-term effects of retirement are replaced by positive ones. Nevertheless, similarly to the former, more men worry about their health all the time (by 20 pp; p<0.001), but they are worried about the problems with their digestive system lest often (by 11 pp; p=.044). Even though more retired men than nonretired declare encountering health problems hampering their everyday living sporadically (by 18 pp; p=.002), less men declare having them often (by 7 pp; p=.073). Furthermore, occurrence of physical problems among retired men is lower (by 15 pp; p=.001), as well as of pain in neck or arms muscles (by 13 pp; p=.027), shortness of breath (by 13 pp; p=.006) and tiredness not related to work (by 14 pp; p=.013). On the other hand, more retired men are troubled by headaches (by 12 pp; p=.032) and problems with sleeping (by 11 pp; p=.006).

## 5. Discussion

The human life cycle is characterized by important and spaced transitions and phases. Each of them is accompanied by various events that modify human behaviour. One of such events is retirement, which often leads to the necessity to reorganize previous lifestyles. Retirement is characterized by changes of biological, psychological, and social character [28] constituting barriers which were often not observed at a younger age [29]. Biological changes (concerning the structure and function of all body organs) lead to deteriorating health and weakening physical strength (decline in fitness and physical capacity) [30]. This obviously limits the performance of many tasks and potentially impacts on the quality of life of older adults [17]. Psychological changes (disturbed emotional and cognitive functioning) [31] lead to a decrease in intellectual performance, deteriorated memory, slower learning, reduced resistance to stress, which makes it difficult to function in a quickly developing civilization. With social changes (related to the retirement, lower income, care for disabled parents or the death of close people), older adults find themselves in new situations and roles [32]. This is accompanied by weakening social bonds (fewer opportunities for establishing social relations, fewer contacts with other people) and, consequently, the risk of isolation from the environment.

Therefore, promotion of the idea of active ageing, defined by Organisation for Economic Co-operation and Development [33] as the ability of a person to live a productive life in society and within the economy as the person becomes older, requires analysis of factors that characterize the lives of older adults. Although the WHO's concept of the quality of life [34] is quite complex with relation to older adults and it covers various aspects (sensory abilities; autonomy; past, present and future activities; social participation; death and dying; and intimacy), one of the important factors is physical/sports activity. This concept is currently being extensively explored and discussed, not only in the context of the correlations with health, but also in the context of developing social resources. The concept is increasingly perceived as an important source of development of personal and social capital, with particular reference to the effects on the labour market and the respective economic consequences. Arguments for this have been provided by numerous researchers, including Lechner [35], Lechner & Sari [36], and Lechner & Downward [37]. They show that regular involvement in sport (especially within sport organizations) can encourage development of problem-free and deep relationships [38, 39]. Therefore, participation in sports clubs may play an important role in helping adults to stay active and to prevent frailty through the increased levels of PA among sports club members [40]. Involvement in sport and recreation activities together with other people, a club-style leisure time, in which an important function is performed by social contacts, provides substantial benefits: included interpersonal benefits (intergenerational opportunities and role models) and organizational benefits (volunteering, financial contributions and maximised facility usage) for engaging older adults [41]. In this sense, S/EA ceases to be limited to the personal interests of older adults and becomes an important social issue and a component of policies [40–42].

Researchers suggest that the problems of the relationships between retirement and participation in S/E are an important and often neglected area [43]. So far, the scientific evidence remains to be inconsistent. Some studies point to a positive relationship [44] of retirement with involvement in PA, while others suggest the opposite tendencies [45]. Little is known about the short- and long-term effects of the retirement [46].

The results of our study, which analysed this research area, show that retirement may have a significant impact on S/EA of older adults. This is particularly true for the long-term perspective of retirement (effects recorded in 2015). Retirement in the short-term perspective (in the period between 2011 and 2013) did not have a significant effect on S/EA (neither in the group of women nor men). This means that Polish older adults continue to rarely undertake PA. This is confirmed by the Eurobarometer survey, according to which regular PA in 2009 was reported by only 8% of Poles aged 55-69 and 5% of those at the age of 70, whereas a relative regularity was found in 33% and 30%, respectively [20]. A survey by Kantar TNS S.A. [47] also showed that the health dose of PA recommended by WHO was met in 2017 by only 8% of Poles aged 60-69. In the short-term perspective, S/EA among the retired men surveyed in our study declined, although insignificantly. However, comparison with the people who did not retire indicates a significant difference in S/EA, meaning that the former were more often active when they worked. Therefore, part of working men, after retiring, stopp being involved in sports or exercising. This is indirectly confirmed by declarations of various S/EA forms. At least one of them (multiple-choice from 10 answers) was declared by 32.4% of nonretired men in 2011, while after the retirement, this percentage amounted in 2013 to only 27.2%. With the short-term perspective concerning the retired women, the percentage increased insignificantly (26.1% and 28.6%, respectively). It is worth emphasizing that the* Social Diagnosis* did not take into account any forms of dance, which could have slightly changed the results (especially in women).

Researchers suggest that PA can increase quite significantly after retirement (intensity decreases while frequency increases) [46]. It seems, however, that in Polish respondents, the fact of retiring (and the related more leisure time) is not a stimulus to increase S/EA. Analysis of health behaviours in men reveals that their health status is also not a sufficient stimulus for them. Perhaps the men were misled by their satisfaction from health status (declared by them after retiring in the period between 2011 and 2013). However, it is debatable why they were not motivated by more often reported serious diseases, nagging health problems that impaired their daily functioning (e.g., digestive ailments, heart palpitations) and the fact that they were constantly worried about their own health. This should encourage some reflection and willingness to take necessary actions. Is it caused by the passivity of men in general during retirement? Perhaps this is the case, since as argued by Sweet et al. [48], the identity of men is closely linked to their professional careers. Retirement is usually associated with the loss of a sense of identity and, therefore, greater passivity, susceptibility to depression, hopelessness and suicide, not only in men, but also in women [49]. Another potential reason can be the lack of knowledge about health benefits of PA. This problem seems to be quite crucial, because scientists believe that motivation for involvement in regular PA and integrating this activity into daily schedules requires some knowledge, experiencing the related benefits and taking actions aimed at eliminating barriers [41, 42, 50]. Knowledge alone is not sufficiently convincing to induce a person to start exercising (especially people who were previously inactive). The belief that PA will satisfy various needs of older adults (for example, those concerning health or emotional needs) and that that it is “obligatory” or pleasant to do should increase the likelihood of approaching it as one of priorities in life.

Although our study does not exclude the possibility of the lack of knowledge among the respondents, it indicates that men tend to prefer staying at home. There is less social activity among them (e.g., participation in volunteer work, lower number of meetings with friends, and being a member of various organizations). This is confirmed by other studies [51, 52]. Milligan et al. [53] argue that older men not only find it harder than women to make friends late in life, they are also less likely to join community-based social groups that tend to be dominated by women. In contrast to women, men also declare lower motivation for life. This is unsurprising, since as demonstrated in a study by Milligan et al. [53], retirees who had more social group memberships following their transition to retirement had better (1) quality of life and (2) objective health. Despite the fact that women in the short term after retirement have a greater lust for life and are more socially active [54], their participation in sports clubs is decreasing [44]. We suppose that this may result from the widespread perception of sports clubs as a natural form of organization but mainly for young people [55]. Adults do not feel like potential members of such clubs [56] and this is perhaps the barrier for increasing S/EA among older adults.

With the long-term perspective (two to three years), the analysis of the effects of retirement reveals a further decline in S/EA among men. Despite their constant worrying about their health status, only 12.0% of men would be involved in at least one form of S/EA in 2015 (after retirement). This is a radical decrease compared to the period of 2011 to 2013. Perhaps it is related to the less frequently felt (in the analysed period) health problems that impair men's everyday functioning (including those related to movement, digestive disorders, and neck and shoulder pain). A man who is convinced of his good health (although this can be only illusory) does not respond and does not need to change his lifestyle, the more so because the change in health-related behaviours is extremely difficult [57], while maintaining these changes is not always successful [58]. The lack of knowledge, unawareness of health benefits of PA, for example its long-term effect on delaying ageing processes, makes the problem even worse [14, 17].

A significant (but modest) decrease (by .8%) of men's membership in sports clubs accompanies negative effect on S/EA. This coincides with negative impact on other social activities of men (i.e., participation in volunteer work, lower number of meetings with acquaintances, and participating in public meetings). From the point of view of building social support networks, planning the rhythm of the day and activation of retired people can be a very important factor in changing lifestyles. It is important to start from anything. The most difficult thing is to leave homes. And this is not easy for Polish men, because they remain to be reluctant to participate in social meetings and contact their friends (except for the workplace). This leads to the conclusion that the workplace should be an environment that activates people who are going to be retired. This can be achieved by establishing senior clubs, or occupational medicine centres, which, with periodical and obligatory preventive examinations have the unique opportunities for including employees in extensive prevention programs, aimed at maintaining good health status, improving its level or active and healthy ageing. This conclusion also applies to Polish women as no significant changes in S/EA were found in this group after retiring in 2015 (26.1% involved in some forms of S/EA in 2011 and 28.3% after retiring in 2015). It should be stressed that as in the case of men, women more often declared satisfaction with their health status and less frequently complained about various health problems (e.g., stomach pains).

## 6. Final Conclusions

The aim of this study was to check how retirement affects S/EA in women and men in Poland. In the context of the cause-and-effect analysis, the interpretation was adopted that this change in the status in the labour market has an effect on the aspects of life described by the outcome variables rather than vice versa. For example, retirement in 2012 or 2013 may affect S/EA in 2013, but starting or stopping S/EA in 2013 is unlikely to lead to retiring or not retiring in 2013 and 2015. Furthermore, it should be noted that short-term effects (recorded in 2013) should be approached with greater caution than medium-term effects (recorded in 2015), because in some situations (related to the design of* Social Diagnosis*, which surveyed the months before the examination), they could have occurred before retirement.

Conclusions resulting from this study indicate the need for preparation of employees for retirement much earlier than in the preretirement age. Employees need to be aware of the importance of prohealth behaviours to healthy ageing (including the need to start S/EA). Knowledge of older adults about such issues continues to be low while current initiatives aimed to improve it (based on the existing system of primary health care and educational campaigns) are ineffective. Therefore, it is necessary to engage other environments in health promotion campaigns, including the work environment and occupational health physicians. Increasing access to sport facilities and making their use easier and improving communication with sports centres, for example through platforms for cooperation with employers and institutions providing classes for older adults, are critical activities of the work environment. An effective solution can be older adults' getting involved in longer projects, for example, learning new skills (e.g., swimming) or those that are aimed to achieve a specific goal (e.g., an outdoor gym or the Olympics for seniors). Obviously, it is also important to monitor physical activity of employees and, depending on the outcomes, to use appropriate programs: (1) maintaining motivation among employees who were physically active before they retired and (2) raising awareness, encouraging physical activity in employees who are physically passive.

Therefore, it seems necessary to extend the standards of preventive care for employees with PA surveys. Periodic employee surveys provide a unique opportunity for verification of its level and, if necessary, education or health promotion among future pensioners [59]. Apart from educating, such interventions should be aimed at establishing a plan of long-term health initiatives, such as modification of diets or increasing PA. The inclusion of people in the preretirement age in such initiatives is of key importance on the general social scale and is justified not only from the epidemiological but also sociodemographic point of view. This is also consistent with the priorities adopted for the years 2013-2020 by the European Agency for Safety and Health at Work (EU-OSHA).* Priorities for occupational safety and health research in Europe: 2013–2020* [60] points to the problem of the ageing population in European countries as the biggest challenge for occupational health care [61].

## Figures and Tables

**Table 1:** Short-run effects of retiring (between April 2011 and April 2013) depending on gender – estimates of ATEs for women and men.

Variable	Men				Women			
	Total number of observations	Retired	Nonretired	P-value	Total number of observations	Retired	Nonretired	P-value
*Sports*								
Sport activity 2013	1424	.223	.252	.439	1186	.317	.284	.340
Member of sports club 2013	1428	.013	.011	.795	1190	<.001	.003	.062
*Social activeness*								
Friends met regularly 2013	1411	4.576	4.798	.657	1181	4.76	4.577	.663
Acquaint. met regularly 2013	**1405**	**4.321**	**5.646**	**.011**	1172	5.637	5.358	.566
Friends 2013	1418	5.747	6.265	.467	1176	6.286	6.594	.530
Work for local society 2011-2013	1428	.138	.189	.130	1190	.177	.135	.133
Member of organisations 2013	**1428**	**.116**	**.200**	**.046**	1190	.165	.165	.991
Took part in public meeting 2013	1428	.196	.200	.920	1190	.100	.142	.095
Voluntary activities 2013	1428	.293	.299	.881	1190	.268	.232	.264
*Attitude*								
Too much alcohol in 2012	**1428**	**.261**	**.102**	**<.001**	**1190**	**<.001**	**.017**	**<.001**
Lust for life 2013	**1427**	**7.954**	**8.379**	**.013**	**1189**	**8.863**	**8.544**	**.021**
Losing interest in sex 2013	1428	.673	.642	.414	1190	.834	.848	.620
Dissatisfaction with sex life 2013	1410	3.586	3.570	.914	1175	4.184	4.192	.958
*Health – general aspects*								
BMI 2013	**1410**	**26.444**	**27.842**	**<.001**	1180	27.763	27.605	.661
Dissatisfaction with health 2013	**1427**	**2.956**	**3.290**	**.007**	1189	3.238	3.394	.110
Significant health worries 2013	**1428**	**.284**	**.185**	**.009**	1190	.166	.183	.585
Health problems often 2013	**1428**	**.237**	**.164**	**.045**	1190	.158	.179	.453
Health problems sometimes 2013	**1428**	**.397**	**.550**	**.001**	1190	.614	.544	.064
Physical problems 2013	1428	.227	.207	.599	1190	.189	.238	.098
Seriously ill 2012	**1428**	**.278**	**.19**	**.022**	**1190**	**.129**	**.189**	**.024**
*Health – specific ailments*								
Problems with sleeping 2013	**1428**	**.045**	**.125**	**.004**	1190	.109	.135	.343
Digestion worries 2013	**1428**	**.234**	**.315**	**.041**	1190	.403	.372	.396
Headaches 2013	1428	.302	.374	.088	1190	.498	.518	.594
Stomach pains 2013	1428	.261	.264	.944	1190	.321	.390	.054
Pain in neck or arm muscles 2013	1428	.489	.574	.055	1190	.523	.566	.265
Chest or heart pains 2013	1428	.368	.324	.291	1190	.274	.335	.080
Dry mouth or throat 2013	1428	.295	.265	.444	1190	.351	.326	.491
Sweating 2013	**1428**	**.170**	**.237**	**.048**	**1190**	**.295**	**.437**	**<.001**
Shortness of breath 2013	1428	.296	.265	.410	1190	.244	.299	.115
Body pains 2013	**1428**	**.464**	**.548**	**.058**	1190	.560	.571	.775
Palpitation 2013	**1428**	**.310**	**.213**	**.012**	**1190**	**.257**	**.325**	**.037**
Shivers or convulsions 2013	1428	.086	.091	.821	1190	.075	.109	.113
Pressure on bladder 2013	1428	.319	.259	.149	1190	.273	.268	.886
Tiredness 2013	1428	.416	.448	.471	1190	.508	.506	.958
Constipation 2013	1428	.060	.107	.087	1190	.253	.238	.627
Nosebleeds 2013	1428	.017	.047	.104	1190	.045	.061	.308
Blood pressure 2013	1428	.371	.345	.550	1190	.418	.402	.665

Notes: bold rows indicate significant differences between the treatment group (i.e., people who were retired in 2013) and the comparison group (average values, constructed based on similar to treated ones, in terms of propensity score and balancing variables, people) at p<.05. The ATEs (effects of retiring) were estimated separately for women and men.

**Table 2:** Middle-run effects of retiring (between April 2013 and April 2015) depending on gender – estimates of ATEs for women and men.

Variable	Men				Women			
	Total number of observations	Retired	Nonretired	P-value	Total number of observations	Retired	Nonretired	P-value
*Sports*								
Sport activity 2015	**924**	**.117**	**.237**	**.012**	782	.236	.264	.494
Member of sports club 2015	**929**	**<.001**	**.008**	**.008**	798	<.001	<.001	.456
*Social activeness*								
Friends met regularly 2015	927	5.200	4.567	.336	796	3.962	4.334	.255
Acquaint. met regularly 2015	**924**	**4.14**	**5.84**	**.015**	787	5.369	4.938	.361
Friends 2015	927	5.92	6.628	.455	796	5.452	6.043	.282
Work for local society 2013-2015	929	.095	.174	.093	798	.178	.143	.235
Member of organisations 2015	929	.073	.174	.125	798	.156	.158	.947
Took part in public meeting 2015	**929**	**.129**	**.247**	**.012**	798	.186	.189	.937
Voluntary activities 2015	929	.196	.295	.059	798	.238	.221	.628
*Attitude*								
Too much alcohol in 2014	929	.042	.092	.076	**798**	**<.001**	**.020**	**<.001**
Lust for life 2015	928	8.144	8.453	.107	796	8.749	8.48	.100
Losing interest in sex 2015	**929**	**.421**	**.696**	**<.001**	**798**	**.942**	**.871**	**.005**
Dissatisfaction with sex life 2015	**920**	**3.928**	**3.559**	**.070**	781	4.517	4.397	.526
*Family life*								
Married 2015	929	.838	.836	.972	798	.632	.686	.209
*Health – general aspects*								
BMI 2015	**926**	**25.52**	**28.027**	**<.001**	**796**	**28.573**	**27.734**	**.044**
Dissatisfaction with health 2015	927	3.290	3.286	.979	**795**	**3.213**	**3.456**	**.027**
Significant health worries 2015	**929**	**.390**	**.184**	**<.001**	798	.151	.191	.257
Health problems often 2015	929	.088	.159	.073	798	.172	.186	.654
Health problems sometimes 2015	**929**	**.722**	**.543**	**.002**	798	.540	.542	.961
Physical problems 2015	**929**	**.056**	**.202**	**.001**	798	.183	.231	.190
Seriously ill 2014	929	.104	.171	.137	798	.231	.190	.241
*Health – specific ailments*								
Problems with sleeping 2015	**929**	**.220**	**.110**	**.006**	798	.132	.111	.531
Digestion worries 2015	**929**	**.236**	**.347**	**.044**	798	.358	.381	.590
Headaches 2015	**929**	**.465**	**.349**	**.032**	798	.480	.511	.498
Stomach pains 2015	929	.215	.288	.184	**798**	**.300**	**.405**	**.015**
Pain in neck or arm muscles 2015	**929**	**.424**	**.552**	**.027**	798	.667	.590	.080
Chest or heart pains 2015	929	.242	.296	.283	**798**	**.394**	**.286**	**.010**
Dry mouth or throat 2015	929	.251	.239	.813	798	.410	.334	.074
Sweating 2015	929	.104	.189	.057	798	.374	.373	.976
Shortness of breath 2015	**929**	**.118**	**.246**	**.006**	798	.296	.284	.778
Body pains 2015	929	.578	.548	.586	**798**	**.665**	**.552**	**.009**
Palpitation 2015	929	.222	.217	.898	**798**	**.368**	**.279**	**.029**
Shivers or convulsions	929	.112	.069	.153	798	.048	.069	.305
Pressure on bladder 2015	929	.176	.236	.256	798	.263	.260	.938
Tiredness 2015	**929**	**.268**	**.409**	**.013**	**798**	**.622**	**.500**	**.007**
Constipation 2015	929	.107	.110	.925	798	.148	.206	.108
Nosebleeds 2015	929	.024	.042	.388	798	.050	.040	.571
Blood pressure 2015	929	.203	.310	.052	798	.462	.381	.068

Notes: bold rows indicate significant differences between the treatment group (i.e., people who were retired in 2013) and the comparison group (average values, constructed based on similar to treated ones, in terms of propensity score and balancing variables, people) at p<.05. The ATEs (effects of retiring) were estimated separately for women and men.

## Data Availability

The data used to support the findings of this study are included within the article.

## Supplementary Materials

Supplementary Materials Annex 1: questions from the Social Diagnosis utilized in the analysis. The annex contains full information on questions and lists of answers from Social Diagnosis questionnaires that have been used in our study. Information on the recoding of the answer to values of the variables is also included (if applicable). Annex 2: descriptive statistics for the outcome and balancing variables. The annex contains information on means and standard deviations of the continuous and categorical variables used in the analysis, as well as ratios for the binary variables.
