# Supplementary material for: Short-Term and Medium-Term Impact of Retirement on Sport Activity, Self-Reported Health, and Social Activity of Women and Men in Poland
Source: Biomed Res Int. 2019 Apr 11;2019:8383540. doi: 10.1155/2019/8383540 (PMC6487168; doi:10.1155/2019/8383540)
Supplement: Supplementary Materials — Annex 1: questions from the Social Diagnosis utilized in the analysis. The annex contains full information on questions and lists of answers from Social Diagnosis questionnaires that have been used in our study. Information on the recoding of the answer to values of the variables is also included (if applicable). Annex 2: descriptive statistics for the outcome and balancing variables. The annex contains information on means and standard deviations of the continuous and categorical variables used in the analysis, as well as ratios for the binary variables. [file 8383540.f1.pdf]

Annex 1. Questions from the Social Diagnosis utilized in the analysis

Variable	Question	Possible answers
Treatment variable: Retirement	Based on Source of income: main and additional	11 permanent paid employment in the public sector 12 permanent paid employment in the private sector 13 temporary paid employment in the public sector 14 temporary paid employment in the private sector 15 use of an agricultural holding 16 helping in an agricultural holding 17 employer outside an individual holding in agriculture 18 permanent work for one's own account (also self-employment) 19 temporary work for one's own account 20 Helping in work for one's own account 21 old age pension (apart from the agricultural social insurance system) 22 old age pensions for individual farmers (under insurance in the Agricultural Social Insurance Fund, KRUS) 23 disability pensions 24 family pensions 25 maternity benefits 26 unemployment benefits 27 other benefits from the Labour Fund 28 allowance for persons on child care leaves (former child care benefits) 29 other social insurance benefits (such as child birth allowance, funeral allowance, sickness allowance) 30 family benefits and allowance in accordance with the Act on Family Benefits of 2003, as amended, housing allowance 31 social assistance benefits 32 other social assistance benefits (such as benefits for persons brining up children, special purpose benefits and extraordinary benefits) 33 children maintenance 34 other income of a social benefit nature (including scholarships)<br>35 income from own property (interest, dividends, etc.) 36 income from the rental of a house, apartment or garage 37 foreign old age and disability pensions 38 benefits under a voluntary sickness and accident insurance system<br>39 compensation under other insurance schemes 40 donations, maintenance from private persons 41 other income 42 other revenues (sale of property, savings, credits) 43 being supported by other household members<br>Recoded as binary variable ((main = (21 or 22) and (additional >20 or empty)) as 1)
Sport activity	Do you practice any sport or physical activity?	1. no, I do not practice any sport or physical activity<br>2. aerobics<br>3. running/jogging/nordic walking<br>4. gym<br>5. cycling<br>6. skiing or other winter sports<br>7. swimming<br>8. football or other team sports

		9. yoga<br>10. martial arts<br>11. another sport or type of physical activity.<br>Recoded as binary variable (2-11 as 1)
Too much alcohol	In the last year, have you drunk too much alcohol	Yes; No
Friends met regularly	How many persons you contact with regularly for social and personal reasons (at least several times a year), among friends?	Open-ended question (integer)
Acquaintances met regularly	How many persons you contact with regularly for social and personal reasons (at least several times a year), among acquaintances (work/school colleagues, neighbors and others)?	Open-ended question (integer)
Friends	How many persons do you consider to be your friends?	Open-ended question (integer)
Work for local society	During the last two years, have you been involved in any actions for the benefit of your local community (gmina, housing estate, town or neighborhood)?	Yes; No
Member of organisations	Are you a member of any organizations, associations, parties, committees, councils, religious groups or clubs?	1. Yes, one; 2. Yes, two; 3. Yes, three or more; 4. No (4 recoded as 0, 1 as 1, 2 as 2, 3 as 3)
Member of a sports club	Do you currently take active part in sports club?	Yes; No
Took part in public meeting	Have you attended any public meeting in the last year (but not at your workplace)?	Yes; No
Voluntary activities	Have you in the last year done any voluntary work for people outside the family or for a social organization?	1. Yes, often; 2. Yes, rarely; 3. No (recoded as a binary variable, 1 as 1, 2 as 1, 3 as 0; for 2011 only 1: Yes and 2: No)
Lust for life	At present, how strong is your willingness to live?	1: I do not want to live at all, 2, 3,..., 10: I want to live very much
Losing interest in sex	Read the four statements in each point carefully and then choose one that describes best your feelings and beliefs during the last month	0. I have not noticed any recent change in my interest in sex; 1. I am less interested in sex than I used to be.; 2. I am much less interested in sex now.; 3. I have lost interest in sex completely. Recoded as a binary variable (1-3 recoded as 1).
Dissatisfaction with sex life	To what extent are you satisfied with your sex life?	1: very satisfied, 2: satisfied, ... 6: very not satisfied, 7: not applicable
BMI	Calculated based on answers to open-ended questions: How tall are you? (cm); What is your weight? (kg)	
Dissatisfaction with health	To what extent are you satisfied with your health condition?	1: very satisfied; 2: satisfied; ... 6: very not satisfied, 7: not applicable
Problems with sleeping	Read the four statements in each point carefully and then choose one that describes best your feelings and beliefs during the last month.	0. I sleep at least as well as I used to.<br>1. I do not sleep as well as I used to.

		2. In the morning, I wake up 1-2 hours earlier and find it difficult to fall asleep again.<br>3. I wake up several hours too early and I can‘t get back to sleep.<br>Recoded as a binary variable (2 and 3 as 1).
Digestion worries	Read the four statements in each point carefully and then choose one that describes best your feelings and beliefs during the last month.	0. I am not worried about my health any more than I used to be.<br>1. I am worried about such ailments as: stomach pains, upset stomach, or constipation.<br>2. I am very worried about my health; I think about it constantly.<br>3. My health condition is so worrying that I cannot think of anything else.<br>Recoded as a binary variable (1 as 1).
Significant health worries	Read the four statements in each point carefully and then choose one that describes best your feelings and beliefs during the last month.	0. I am not worried about my health any more than I used to be.<br>1. I am worried about such ailments as: stomach pains, upset stomach, or constipation.<br>2. I am very worried about my health; I think about it constantly.<br>3. My health condition is so worrying that I cannot think of anything else.<br>Recoded as a binary variable (2 and 3 as 1).
Health problems often	In the recent months your health problems have made it difficult for you to perform everyday activities or to take part in other activities?	1. Often<br>2. Once or twice<br>3. Never<br>Recoded as a binary variable (1 as 1)
Health problems sometimes	In the recent months your health problems have made it difficult for you to perform everyday activities or to take part in other activities?	1. Often<br>2. Once or twice<br>3. Never<br>Recoded as a binary variable (2 as 1)
Physical problems	In the recent months you have suffered from ailments, such as bones aching or shortness of breath, etc., which has made it difficult for you to leave home, climb the stairs, etc?	1. Often<br>2. Once or twice<br>3. Never<br>Recoded as a binary variable (1 as 1)
Seriously ill	In the last year have you been seriously ill?	Yes; No
Headaches	Below you will find a list of some ailments. Please specify whether you suffered from them LAST MONTH: strong headaches	1. I did not suffer.<br>2. I suffered for less than 15 fays<br>3. I suffered at least for half a month<br>Recoded as a binary variable (2 and 3 as 1)
Stomach pains	(as above): stomach pains or flatulence	as above

Pain in neck or arm muscles	(as above): pain or tension in the neck or arm muscles	as above
Chest or heart pains	(as above): chest or heart pains	as above
Dry mouth or throat	(as above): dry mouth or throat	as above
Sweating	(as above): attacks of excessive sweating	as above
Shortness of breath	(as above): shortness of breath	as above
Body pains	(as above): pains throughout the whole body	as above
Palpitation	(as above): accelerated heartbeat (palpitation)	as above
Shivers or convulsions	(as above): shivers or convulsions	as above
Pressure on bladder	(as above): pressure on the bladder and more frequent urinating	as above
Tiredness	(as above): a feeling tiredness not associated with work	as above
Constipation	(as above): constipation	as above
Nosebleeds	(as above): nosebleeds	as above
Blood pressure	(as above): sudden changes of blood pressure	as above
Age	Pre-coded in the database – based on date of birth	
Years of education completed	Pre-coded in the database – based on direct question	
Higher Education	Based on information on educational attainment pre-coded in the database (1 if higher education with at least a PhD title, higher education with at least an MA degree or an equivalent degree, higher education with an Engineer or Bachelor degree)	
Number of adults living in the same household	Calculated based on number of people living in the household at age of at least 20[1]	
Living in a large city	Dummy for cities with more than 500,000 inhabitants (pre-coded)	
Living in a rural area	Dummy for rural areas (pre-coded)	
Disability	Disability	1 for the persons who have a valid certificate from the Social Insurance Institution (ZUS);<br>2 for the persons who have a valid certificate from the Disability Evaluation Board at the Poviat Centre<br>of Family Support (ZOoN at PCPR);<br>3 for the persons who have a valid certificate from the Social Insurance Institution and ZOoN at PCPR;<br>4 for the persons who have stated that due to disability or disease they have completely or partly<br>limited ability to perform such activities as learning, working or taking care of own household but they<br>do not have a certificate from the medical board;<br>5 disability of children aged below 16;<br>0 other cases;<br>8 not applicable (the person is not a disabled person)<br>Recoded as a binary variable (0-5 as 1)
Marital status	Marital status	1 unmarried<br>2 married<br>3 widow(er)<br>4 divorced<br>5 legally separated (based on a court decision)

[1] Even though 18 is age of maturity in Poland, 19 has been chosen as the highest age for 'non-adults', since most of the high schools students graduate during the summer in a year in which they are 19 years old.

		6 practically separated (the spouses do not live together without a court decision).<br>Recoded as binary (2 as 1)
Permanent employment	What is the type of work this person performs at his/her main job?	1. based on an employment contract for a specified period of time (apart from the contracts listed below, being non-standard forms of employment (6-11), and for a period longer than one year)<br>2. based on an employment contract for an unspecified period of time<br>3. self-employed entrepreneur hiring employers<br>4. self-employed<br>5. helping in a family business without pay<br>6. temporary job (based on fixed-term employment contracts, such as replacement contracts, contracts for specific work)<br>7. other short-term contracts (such as summer traineeships, employment contracts for a period shorter than one year)<br>8. trial period employment<br>9. paid employment on the basis of a civil law contract (contract of mandate, contract for specific work)<br>10. paid employment without a formal contract or with an oral agreement<br>11. other<br>Recoded as binary (2 as 1)
Inactive	During the last 4 weeks, has this person been seeking a job or a different job?	1 YES and I am currently unemployed<br>2 YES and I am currently employed<br>3 NO and I am currently unemployed but I have already found a job<br>4 NO and I am currently unemployed<br>5 NO and I am currently employed<br>Recoded as binary (4 as 1)
Higher education of father	What was the educational attainment of your father (or main guardian) when you were 14?	1 primary not completed<br>2 primary<br>3 vocational<br>4 secondary not completed<br>5 secondary vocational<br>6 secondary general<br>7 higher not completed (including post-secondary)<br>8 higher

		9 I do not know.<br>Recoded as binary (7 and 8 as 1).
General trust	In general, do you believe that most people can be trusted or are you of the opinion that one can never be too careful with people?	1 most people can be trusted<br>2 one cannot be too careful in dealing with people<br>3 it is hard to say<br>Recoded as binary (1 as 1).
Trust towards Parliament	Do you trust: Sejm	1 yes<br>2 no<br>3 I have no opinion<br>Recoded as binary (1 as 1).
Health benefits	In the last year, have you used the services of healthcare units paid for by the employer (under a medical services plan or health insurance)?	Yes; No
Insomnia	Read the four statements in each point carefully and then choose one that describes best your feelings and beliefs during the last month	0 I have not experienced any change in my sleeping pattern.<br>1 I do not sleep as well as I used to.<br>2 In the morning, I wake up 1-2 hours earlier and find it difficult to fall asleep again.<br>3 I wake up several hours too early and I can't get back to sleep.<br>Recoded as binary variable (2-3 recoded as 1).
High energy	Please specify to what extent these statements match your beliefs and attitudes:<br>I have a lot of energy.	1 Definitely yes<br>2 Yes<br>3 Rather yes<br>4 Neither yer nor not<br>5 Rather not<br>6 No<br>7 Definitely not<br>Recoded as binary (1,2 and 3 as 1)

Notes: Based on Social Diagnosis, questionnaires. www.diagnoza.com [downloaded 19.03.2017].

Annex 2. Descriptive statistics for the outcome and balancing variables

Variable	Year	Treated men	Non-treated men	Treated women	Non-treated women
Sport activity	2011	0.324 (179)	0.258 (2,298)	0.261 (249)	0.274 (2,311)
	2013	0.272 (187)	0.272 (2,321)	0.286 (258)	0.297 (2,323)
	2015	0.268 (108)	0.235 (1,542)	0.283 (180)	0.277 (1,502)
Too much alcohol	2011	0.090 (209)	0.109 (2,679)	0.007 (275)	0.016 (2,455)
	2013	0.027 (187)	0.070 (2,331)	0.000 (258)	0.013 (2,329)
	2015	0.046 (108)	0.107 (1,555)	0.000 (184)	0.021 (1,533)
Friends met regularly	2011	4.850 ± 4.512 (174)	5.098 ± 5.280 (2,287)	4.863 ± 5.283 (249)	4.873 ± 4.985 (2,298)
	2013	5.423 ± 5.933 (184)	4.912 ± 5.624 (2,305)	4.714 ± 5.349 (256)	4.511 ± 4.420 (2,306)
	2015	5.574 ± 5.595 (108)	4.747 ± 5.059 (1,547)	4.429 ± 4.007 (184)	4.411 ± 4.125 (1,525)
Acquaintances met regularly	2011	6.28 ± 6.105 (175)	6.145 ± 7.229 (2,260)	5.654 ± 5.999 (246)	5.757 ± 7.058 (2,264)
	2013	5.673 ± 6.320 (184)	5.964 ± 7.570 (2,290)	5.388 ± 6.442 (250)	5.358 ± 5.915 (2,292)
	2015	5.308 ± 5.457 (107)	6.125 ± 8.070 (1,544)	4.927 ± 5.460 (180)	5.260 ± 5.924 (1,514)
Friends	2011	7.717 ± 7.449 (177)	7.042 ± 7.827 (2,286)	7.584 ± 8.109 (248)	6.588 ± 6.707 (2,306)
	2013	7.194 ± 8.239 (185)	6.278 ± 6.518 (2,305)	6.883 ± 6.854 (258)	6.061 ± 5.577 (2,303)
	2015	7.222 ± 7.660 (108)	6.340 ± 6.900 (1,549)	6.721 ± 7.043 (183)	6.033 ± 5.698 (1,529)
Work for local society	2011	0.186 (209)	0.181 (2,679)	0.174 (275)	0.158 (2,455)
	2013	0.155 (187)	0.188 (2,331)	0.151 (258)	0.151 (2,329)
	2015	0.204 (108)	0.181 (1,555)	0.125 (184)	0.147 (1,533)
Member of organisations	2011	0.210 ± 0.522 (209)	0.214 ± 0.555 (2,679)	0.185 ± 0.473 (275)	0.192 ± 0.481 (2,455)
	2013	0.155 ± 0.443 (187)	0.216 ± 0.557 (2,331)	0.155 ± 0.466 (258)	0.177 ± 0.493 (2,329)
	2015	0.185 ± 0.566 (108)	0.197 ± 0.523 (1,555)	0.130 ± 0.411 (184)	0.179 ± 0.485 (1,533)
Member of a sports club	2013	0.005 (187)	0.014 (2,331)	0.000 (258)	0.004 (2,329)
	2015	0.000 (108)	0.015 (1,555)	0.000 (184)	0.002 (1,533)
Took part in public meeting	2011	0.301 (209)	0.281 (2,679)	0.229 (275)	0.231 (2,455)
	2013	0.176 (187)	0.203 (2,331)	0.120 (258)	0.136 (2, 329)
	2015	0.213 (108)	0.253 (1,555)	0.179 (184)	0.180 (1,533)
Voluntary activities	2011	0.248 (209)	0.223 (2,679)	0.152 (275)	0.186 (2,455)
	2013	0.273 (187)	0.319 (2,331)	0.205 (258)	0.249 (2,329)
	2015	0.278 (108)	0.324 (1,555)	0.228 (184)	0.256 (1,533)
Lust for life	2011	8.335 ± 1.851 (179)	8.369 ± 1.787 (2,313)	8.488 ± 1.917 (252)	8.447 ± 1.853 (2,323)
	2013	8.283 ± 1.857 (187)	8.367 ± 1.820 (2,329)	8.511 ± 1.914 (258)	8.489 ± 1.800 (2,325)
	2015	8.601 ± 1.605 (108)	8.424 ± 1.723 (1,554)	8.491 ± 1.845 (183)	8.564 ± 1.678 (1,530)
Losing interest in sex	2011	0.770 (209)	0.606 (2,679)	0.872 (275)	0.789 (2,455)
	2013	0.781 (187)	0.592 (2,331)	0.849 (258)	0.804 (2,329)
	2015	0.750 (108)	0.648 (1,555)	0.918 (184)	0.830 (1,533)
Dissatisfaction with sex life	2011	3.516 ± 1.747 (178)	3.248 ± 1.614 (2,298)	4.270 ± 2.023 (251)	3.743 ± 1.932 (2,295)
	2013	3.729 ± 1.754 (185)	3.427 ± 1.684 (2,313)	4.445 ± 2.074 (256)	3.891 ± 1.963 (2,303)
	2015	3.588 ± 1.715 (107)	3.458 ± 1.697 (1,548)	4.782 ± 2.108 (184)	4.069 ± 2.000 (1,515)
Marital status	2011	0.861 (209)	0.819 (2,679)	0.690 (275)	0.721 (2,455)
	2013	0.871 (187)	0.818 (2,331)	0.667 (258)	0.712 (2,329)

	2015	0.852 (108)	0.829 (1,555)	0.636 (184)	0.699 (1,533)
BMI	2011	27.91 ± 4.833 (176)	27.59 ± 4.176 (2,287)	27.91 ± 4.935 (250)	26.93 ± 4.693 (2,310)
	2013	27.724 ± 4.347 (184)	27.723 ± 4.195 (2,297)	27.47 ± 4.814 (257)	27.077 ± 4.68 (2,302)
	2015	27.81 ± 3.972 (108)	27.87 ± 4.120 (1,547)	27.71 ± 4.497 (184)	27.44 ± 4.914 (1,520)
Dissatisfaction with health	2011	3.547 ± 1.333 (179)	3.176 ± 1.264 (2,311)	3.386 ± 1.289 (251)	3.251 ± 1.241 (2,321)
	2013	3.326 ± 1.358 (187)	3.202 ± 1.260 (2,327)	3.404 ± 1.307 (257)	3.272 ± 1.256 (2,324)
	2015	3.25 ± 1.238 (108)	3.208 ± 1.248 (1,552)	3.393 ± 1.212 (183)	3.308 ± 1.228 (1,527)
Problems with sleeping	2011	0.157 (209)	0.099 (2,679)	0.178 (275)	0.098 (2,455)
	2013	0.144 (187)	0.116 (2,331)	0.178 (258)	0.112 (2,329)
	2015	0.148 (108)	0.091 (1,555)	0.168 (184)	0.101 (1,533)
Digestion worries	2011	0.258 (209)	0.256 (2,679)	0.327 (275)	0.315 (2,455)
	2013	0.272 (187)	0.309 (2,331)	0.360 (258)	0.359 (2,329)
	2015	0.333 (108)	0.336 (1,555)	0.336 (184)	0.346 (1,533)
Significant health worries	2011	0.210 (209)	0.130 (2,679)	0.178 (275)	0.168 (2,455)
	2013	0.240 (187)	0.151 (2,331)	0.205 (258)	0.168 (2,329)
	2015	0.148 (108)	0.149 (1,555)	0.206 (184)	0.165 (1,533)
Health problems often	2011	0.196 (209)	0.122 (2,679)	0.152 (275)	0.147 (2,455)
	2013	0.208 (187)	0.138 (2,331)	0.186 (258)	0.157 (2,329)
	2015	0.120 (108)	0.135 (1,555)	0.152 (184)	0.157 (1,533)
Health problems sometimes	2011	0.444 (209)	0.439 (2,679)	0.501 (275)	0.496 (2,455)
	2013	0.502 (187)	0.534 (2,331)	0.569 (258)	0.549 (2,329)
	2015	0.611 (108)	0.531 (1,555)	0.559 (184)	0.536 (1,533)
Physical problems	2011	0.248 (209)	0.154 (2,679)	0.174 (275)	0.202 (2,455)
	2013	0.240 (187)	0.177 (2,331)	0.213 (258)	0.217 (2,329)
	2015	0.194 (108)	0.181 (1,555)	0.211 (184)	0.196 (1,533)
Seriously ill	2011	0.205 (209)	0.130 (2,679)	0.200 (275)	0.147 (2,455)
	2013	0.240 (187)	0.155 (2,331)	0.151 (258)	0.160 (2,329)
	2015	0.203 (108)	0.151 (1,555)	0.184 (184)	0.153 (1,533)
Headaches	2011	0.267 (209)	0.274 (2,679)	0.385 (275)	0.421 (2,455)
	2013	0.358 (187)	0.354 (2,331)	0.496 (258)	0.499 (2,329)
	2015	0.324 (108)	0.344 (1,555)	0.483 (184)	0.483 (1,533)
Stomach pains	2011	0.153 (209)	0.208 (2,679)	0.287 (275)	0.328 (2,455)
	2013	0.267 (187)	0.261 (2,331)	0.360 (258)	0.379 (2,329)
	2015	0.342 (108)	0.274 (1,555)	0.353 (184)	0.384 (1,533)
Pain in neck or arm muscles	2011	0.425 (209)	0.381 (2,679)	0.396 (275)	0.407 (2,455)
	2013	0.550 (187)	0.541 (2,331)	0.573 (258)	0.554 (2,329)
	2015	0.537 (108)	0.535 (1,555)	0.635 (184)	0.574 (1,533)
Chest or heart pains	2011	0.258 (209)	0.226 (2,679)	0.236 (275)	0.223 (2,455)
	2013	0.331 (187)	0.292 (2,331)	0.313 (258)	0.278 (2,329)
	2015	0.287 (108)	0.276 (1,555)	0.347 (184)	0.270 (1,533)
Dry mouth or throat	2011	0.234 (209)	0.197 (2,679)	0.218 (275)	0.225 (2,455)
	2013	0.251 (187)	0.256 (2,331)	0.341 (258)	0.305 (2,329)
	2015	0.277 (108)	0.237 (1,555)	0.342 (184)	0.298 (1,533)
Sweating	2011	0.200 (209)	0.159 (2,679)	0.283 (275)	0.318 (2,455)
	2013	0.171 (187)	0.212 (2,331)	0.368 (258)	0.434 (2,329)
	2015	0.175 (108)	0.199 (1,555)	0.353 (184)	0.425 (1,533)
Shortness of breath	2011	0.200 (209)	0.164 (2,679)	0.225 (275)	0.216 (2,455)
	2013	0.267 (187)	0.230 (2,331)	0.317 (258)	0.269 (2,329)
	2015	0.203 (108)	0.230 (1,555)	0.331 (184)	0.283 (1,533)
Body pains	2011	0.397 (209)	0.380 (2,679)	0.418 (275)	0.400 (2,455)
	2013	0.550 (187)	0.522 (2,331)	0.585 (258)	0.535 (2,329)
	2015	0.620 (108)	0.518 (1,555)	0.641 (184)	0.523 (1,533)
Palpitation	2011	0.177 (209)	0.151 (2,679)	0.214 (275)	0.225 (2,455)
	2013	0.240 (187)	0.196 (2,331)	0.267 (258)	0.277 (2,329)
	2015	0.175 (108)	0.195 (1,555)	0.309 (184)	0.259 (1,533)
Shivers or convulsions	2011	0.076 (209)	0.053 (2,679)	0.065 (275)	0.066 (2,455)
	2013	0.080 (187)	0.088 (2,331)	0.108 (258)	0.088 (2,329)

	2015	0.064 (108)	0.066 (1,555)	0.065 (184)	0.073 (1,533)
	2011	0.191 (209)	0.155 (2,679)	0.167 (275)	0.182 (2,455)
Pressure on bladder	2013	0.326 (187)	0.241 (2,331)	0.271 (258)	0.244 (2,329)
	2015	0.324 (108)	0.215 (1,555)	0.277 (184)	0.251 (1,533)
	2011	0.358 (209)	0.311 (2,679)	0.378 (275)	0.372 (2,455)
Tiredness	2013	0.465 (187)	0.425 (2,331)	0.500 (258)	0.481 (2,329)
	2015	0.398 (108)	0.401 (1,555)	0.516 (184)	0.495 (1,533)
	2011	0.100 (209)	0.064 (2,679)	0.123 (275)	0.171 (2,455)
Constipation	2013	0.122 (187)	0.099 (2,331)	0.217 (258)	0.222 (2,329)
	2015	0.120 (108)	0.099 (1,555)	0.206 (184)	0.200 (1,533)
	2011	0.047 (209)	0.034 (2,679)	0.061 (275)	0.038 (2,455)
Nosebleeds	2013	0.058 (187)	0.049 (2,331)	0.042 (258)	0.045 (2,329)
	2015	0.064 (108)	0.038 (1,555)	0.038 (184)	0.039 (1,533)
	2011	0.277 (209)	0.201 (2,679)	0.276 (275)	0.242 (2,455)
Blood pressure	2013	0.374 (187)	0.303 (2,331)	0.395 (258)	0.331 (2,329)
	2015	0.342 (108)	0.286 (1,555)	0.418 (184)	0.352 (1,533)
Age	2011	60.79 ± 3.473 (209)	54.81 ± 4.264 (2,679)	59.38 ± 3.160 (275)	53.81 ± 3.930 (2,455)
Years of education completed	2011	10.85 ± 2.682 (207)	11.55 ± 3.221 (2,640)	11.10 ± 3.000 (266)	11.86 ± 2.886 (2,429)
Higher Education	2011	0.081 (209)	0.101 (2,679)	0.109 (275)	0.127 (2,455)
Number of adults living in the same household	2011	2.990 ± 1.263 (209)	3.417 ± 1.422 (2,679)	2.974 ± 1.349 (275)	3.345 ± 1.463 (2,455)
Living in a large city	2011	0.076 (209)	0.067 (2,679)	0.072 (275)	0.074 (2,455)
Living in a rural area	2011	0.468 (209)	0.505 (2,679)	0.48 (275)	0.444 (2,455)
Disability	2011	0.291 (209)	0.215 (2,679)	0.24 (275)	0.174 (2,455)
Permanent employment	2011	0.267 (209)	0.378 (2,679)	0.149 (275)	0.374 (2,455)
Inactive	2011	0.411 (209)	0.191 (2,679)	0.48 (275)	0.304 (2,455)
Higher education of father	2011	0.014 (209)	0.029 (2,679)	0.029 (275)	0.040 (2,455)
General trust	2011	0.162 (209)	0.113 (2,679)	0.12 (275)	0.135 (2,455)
Trust towards Parliament	2011	0.186 (209)	0.116 (2,679)	0.203 (275)	0.123 (2,455)
Health benefits	2011	0.043 (209)	0.027 (2,679)	0.003 (275)	0.032 (2,455)
High energy	2011	0.411 (209)	0.507 (2,679)	0.44 (275)	0.547 (2,455)

Note: For continuous/categorical variables mean ± standard deviation is shown, for binary variables – a frequency of positive answers. In both cases n (number of non-missing observations) – follows in brackets.
